# Extract from the Proceedings of the Mississippi State Dental Association

**Published:** 1876-10

**Authors:** 


### EXTRACT FROM THE PROCEEDINGS OF THE MISSISSIPPI STATE DENTAL ASSOCIATION.

The second annual meeting of the Mississippi State Dental Association was held in Jackson, Miss., August 16, 1876, at the dental rooms of Dr. A. H. Hilzheim, Dr. J. D. Miles President, in the chair.

The meeting was called to order at 11 o'clock a. m.

The minutes of the last meeting having been read, the Executive Committee reported the following names for membership:

Dr. W. T. Martin, Yazoo City, Miss.

Dr. R. J. Miller, Raymond, Miss.

Dr. G. W. Rembert, Fayette, Miss.

Dr. T. B. Payne, Canton, Miss.

Dr. E. E. Spinks, Meridian, Miss.

A ballot having been taken, the above named gentlemen were declared duly elected.

The President delivered the following annual address:

Companions: I rise with pleasure to greet you on this anniversary of our Dental Association, and extend to all a cordial welcome here. I doubt not, we shall be benefitted in discussing the many interesting subjects offered for our consideration and by friendly interchange of views on the leading topics so ably handled by other kindred associations. When we first met as an Association, we could do little better than organize; now a fair field opens before us, and we should all come square up to the work, There is much for us to do, if we desire to see the status of our profession elevated to that high position so nobly attained in other States. It is true we of Mississippi have had much to contend against in the past. Charlatanism is bold and defiant throughout the State, and why? Simply because it has no organized foe to fight. While I am bitterly opposed to quackery, believe me, Companions, I would not knowingly infringe upon the rights of the most humble operator. But with the advantages of common schools, the aid of Dental and Medical Colleges throughout the land, no one of ordinary capacity has any excuse to practice dentistry without being thoroughly qualified for so responsible a position.

Let us imitate the worthy examples of Dental Associations in other States in procuring the enactment of laws for regulating the practice of dentistry, whose beneficial influence will be felt alike by the dentists and the people. It is for you to say whether we shall plod along in the future as we have done during the past quarter century, or with united efforts enable dentistry to reach and maintain that lofty and dignified position to which it aspires.

In conclusion, let me beg you, in all our deliberations, to exercise charity toward one another, ever remembering that none are perfect, and that all have much to learn. I will only add that I confidently hope that this Association will in future grow in numbers, strength and usefulness, until it shall proudly take its place in the constallation of dental societies of these United States, and shine as brightly as the brightest.

Dr. Payne next introduced the subject of "replanting teeth" and gave the history of several cases in which he had met with great success. Dr. Hilzheim followed Dr. Payne on the same subject and mentioned one case in which eminent success had crowned the operation, the only case he had attempted. The meeting then adjourned until half-past three.

EVENING SESSION.

Dr. Martin exhibited an inter-dental splint which he had used with entire success in a case of fracture of the inferior maxilla. Dr. Jones and Dr. Payne, each mentioned cases where they had used a similar contrivance,

The meeting, on motion of Dr. Hilzheim, adjourned to visit the Lunatic Asylum.

NIGHT SESSION.

The following officers were then elected to serve for the ensuing year:

President, Dr. J. B. Askew, of Vicksburg.

Vice-President, Dr. T. B. Payne, of Canton.

Secretary and Treasurer, Dr. A. Riser, of Port Gibson.

The following delegates were elected to the American Dental Association:

Drs. A. H. Hilzheim and T. W. Martin.

After the appointment of the following committees, the Convention adjourned, to meet at Canton, Miss., the third Wednesday in August, 1877.

Executive Committee—Drs. J. B. Askew, J, D. Miles, R.J. Miller.

Committee to secure the passage of a dental law through the Legislature—Drs. A. H. Hilzheim, J. D. Miles, R. J. Miller.

Committtee of Arrangements—Drs. A. H. Hilzheim, T. B. Payne, W. T. Martin.

